# Minimally invasive colorectal cancer surgery: an observational study of medicare advantage and fee-for-service beneficiaries

**DOI:** 10.1007/s00464-024-11168-0

**Published:** 2024-08-19

**Authors:** Emna Bakillah, James Sharpe, Chris Wirtalla, Drew Goldberg, Maria S. Altieri, Cary B. Aarons, Luke J. Keele, Rachel R. Kelz

**Affiliations:** 1 Department of Surgery, Perelman School of Medicine of the University of Pennsylvania, 3400 Spruce St, 4 Maloney, Philadelphia, PA 19104 USA; 2 Department of Surgery, Center for Surgery and Health Economics, Philadelphia, PA USA; 3 Leonard Davis Institute of Health Economics, University of Pennsylvania, Philadelphia, PA USA; 4 Department of Surgery, Columbia University, New York, NY USA

**Keywords:** Colorectal cancer surgery, Minimally invasive surgery, Medicare advantage, Medicare fee-for-service

## Abstract

**Background:**

Enrollment of Medicare beneficiaries in medicare advantage (MA) plans has been steadily increasing. Prior research has shown differences in healthcare access and outcomes based on Medicare enrollment status. This study sought to compare utilization of minimally invasive colorectal cancer (CRC) surgery and postoperative outcomes between MA and Fee-for-Service (FFS) beneficiaries.

**Methods:**

A retrospective cohort study of beneficiaries  ≥ 65.5 years of age enrolled in FFS and MA plans was performed of patients undergoing a CRC resection from 2016 to 2019. The primary outcome was operative approach, defined as minimally invasive (laparoscopic) or open. Secondary outcomes included robotic assistance, hospital length-of-stay, mortality, discharge disposition, and hospital readmission. Using balancing weights, we performed a tapered analysis to examine outcomes with adjustment for potential confounders.

**Results:**

MA beneficiaries were less likely to have lymph node (12.9 vs 14.4%, *p* < 0.001) or distant metastases (15.5% vs 17.0%, *p* < 0.001), and less likely to receive chemotherapy (6.2% vs 6.7%, *p* < 0.001), compared to FFS beneficiaries. MA beneficiaries had a higher risk-adjusted likelihood of undergoing laparoscopic CRC resection (OR 1.12 (1.10–1.15), *p* < 0.001), and similar rates of robotic assistance (OR 1.00 (0.97–1.03), *p* = 0.912), compared to FFS beneficiaries. There were no differences in risk-adjusted length-of-stay (*β* coefficient 0.03 (− 0.05–0.10), *p* = 0.461) or mortality at 30-60-and 90-days (OR 0.99 (0.95–1.04), *p* = 0.787; OR 1.00 (0.96–1.04), *p* = 0.815; OR 0.98 (0.95–1.02), *p* = 0.380). MA beneficiaries had a lower likelihood of non-routine disposition (OR 0.77 (0.75–0.78), *p* < 0.001) and readmission at 30-60-and 90-days (OR 0.76 (0.73–0.80), *p* < 0.001; OR 0.78 (0.75–0.81), *p* < 0.001; OR 0.79 (0.76–0.81), *p* < 0.001).

**Conclusions:**

MA beneficiaries had less advanced disease at the time of CRC resection and a greater likelihood of undergoing a laparoscopic procedure. MA enrollment is associated with improved health outcomes for elderly beneficiaries undergoing operative treatment for CRC.

**Supplementary Information:**

The online version contains supplementary material available at 10.1007/s00464-024-11168-0.

Colorectal cancer (CRC) is the third most common cancer worldwide, accounting for approximately 10% of all cancer cases, and is most prevalent in people over 50 years of age[1]. Treatment of CRC is multimodal and can include systemic chemotherapy, radiation therapy, immunotherapy, and surgical resection [2]. Surgical treatment of CRC includes tumor resection and removal of surrounding lymph nodes [3]. The technical approaches to surgical resection vary and include open, laparoscopic, and robotic techniques [4].

Prior studies have suggested that laparoscopic surgery is preferred over an open approach for elderly patients with CRC [4]. In a meta-analysis of patients over 65 years of age by Fujii et al., short-term outcomes including estimated blood loss, postoperative complications, and overall morbidity were favorable in patients who received laparoscopic surgery for the treatment of CRC compared to open surgery [5]. Additionally, several studies have indicated that patients over the age of 80 years old can safely undergo laparoscopic CRC resection with similar postoperative outcomes compared to younger patients [6, 7]. Robotic surgery for CRC has also been shown to have comparable outcomes to laparoscopic and open techniques, although the robotic approach is associated with longer operative times and significantly higher costs [8–10].

Medicare advantage (MA) enrollment has been steadily increasing, with approximately half of all eligible Medicare beneficiaries enrolled in MA plans as of 2023 [11, 12]. MA differs from traditional Medicare Fee-for-Service (FFS) in that MA plans have a fixed payment for the total cost of care, which incentivizes MA plans to use managed care techniques to limit their costs [13]. As the population of CRC patients is aging and MA plans are becoming increasingly utilized, it is important to understand the clinical implications managed care plans may have on access to cancer treatment as well as quality of care and outcomes.

When compared to FFS, MA plans are associated with higher use of preventative care visits [14, 15], fewer hospital admissions [16–18] and readmissions [19], shorter hospital length-of-stay [20, 21], lower rates of in-hospital mortality [22], and lower health care spending [17, 23]. On the other hand, FFS enrollees are more likely to receive care at the highest-rated hospitals for cancer care and highest-quality skilled nursing facilities [24–26]. The literature is sparse with regards to the association between Medicare plans and oncologic surgical care. Thus, the aim of this study was to compare utilization of minimally invasive CRC resection as well as postoperative outcomes between MA and FFS beneficiaries.

## Materials and methods

### Data source and population

This is a retrospective cohort study of adults age ≥ 65.5 years of age who underwent colorectal cancer (CRC) resection and were enrolled in Medicare Fee-for-Service (FFS) or Medicare Advantage (MA) from 2016 and 2019. The US Centers for Medicare and Medicaid Services Inpatient, Outpatient, Carrier (Part B), and Durable Medical Equipment files for both Medicare Encounter and Medicare Fee-for-Service claims were used for this study. A 6-month look-back period was used to confirm the index encounter and gather information regarding comorbidities.

CRC diagnoses were identified using International Classification of Diseases, Tenth Revision (ICD-10-CM) codes and tumor location was defined (colon, rectosigmoid, or rectal). (Supplementary Table 1) CRC operations were identified using Current Procedural Terminology (CPT) codes and were designated a procedure type (partial colectomy, total colectomy, large bowel procedure or proctectomy). (Supplementary Table 2) Beneficiaries were classified by their Medicare plan: FFS or MA.

### Covariates

Covariates abstracted directly from the database included: age, sex, race (White, Black, Asian, American Indian or Alaska Native, unknown, or other), Hispanic ethnicity, dual-eligibility status (having both Medicare and Medicaid coverage, as a proxy for socioeconomic status), Elixhauser comorbidity conditions, multimorbid status, claims-based frailty index (CFI), use of multimodal therapy (chemotherapy or radiation therapy), presence of metastases (lymph node or distant), and access type (emergent/urgent or elective).

The Elixhauser comorbidity index is a tool using a comprehensive set of 31 comorbidity measures that are important predictors of outcomes and a weighting algorithm based on the association between comorbidity and death with scoring based on a dichotomous score given to each comorbidity category [27]. The CFI is a composite score based on a patient’s mobility, use of skilled nursing care, and activities of daily living that has been validated in Medicare data [28].

### Outcomes

The primary outcome included operative approach, defined as minimally invasive (laparoscopic) or open using CPT procedure codes. (Supplementary Table 2) Secondary outcomes included robotic assistance (CPT code S2900 [29] and ICD-10-PCS codes 8E0W0CZ, 8E0W3CZ, 8E0W4CZ, 8E0W7CZ, 8E0W0CZ, and 8E0W8CZ [30]), hospital length-of-stay, mortality (30-day, 60-day, and 90-day), discharge disposition (routine defined as ‘home without services’ vs non-routine defined as discharge other than a ‘home without services’), and hospital readmission rate (30-day, 60-day, and 90-day).

### Statistical approach

Descriptive statistics and univariate analyses were performed using chi square test, Wilcoxon rank sum test, and Fisher’s exact test as appropriate. We controlled for observed confounders using balancing weights and a tapered analysis to understand how different adjustment sets affect the results [31–34].

To account for differences in patient characteristics between MA and FFS beneficiaries that could account for estimated differences in outcome, we used balancing weights. Balancing weights are a computational generalization of inverse propensity score weight that we use to create patient populations with similar covariate distributions [35]. Approximate balancing weights assign a scalar weight to each FFS beneficiary such that the reweighted FFS beneficiary satisfy a set of balance constraints that ensure that differences between MA and FFS beneficiaries are small.

Six tapered groups specification were constructed, with each successive specification controlling for additional covariates. The first taper group was patient demographics which included age, sex, and race. The second taper group was socioeconomic status which included dual-eligibility status. The third taper group was baseline medical status which included Elixhauser comorbidity index, CFI, and multimorbid status. The fourth taper group was cancer characteristics which included tumor location, metastases, multimodal therapy, and procedure type. The fifth taper group was access type (emergent/urgent vs elective). The sixth and final taper was controlled for hospitals. To check for balance, we assessed standardized mean differences with an absolute value of < 0.10 indicating non-significance between the two groups. In the models examining postoperative outcomes, we further adjusted for the operative approach.

The study protocol was reviewed and approved by the Institutional Review Board (IRB) at the University of Pennsylvania. The study was performed in accordance with guidelines set forth by the Strengthening the Reporting of Observational Studies in Epidemiology (STROBE) initiative [36].

## Results

### Descriptive statistics

The study population included 148,869 beneficiaries who underwent CRC resection. Of these beneficiaries, 53,023 (35.6%) were enrolled in MA and 95,846 (64.4%) were enrolled in traditional FFS. On average, MA beneficiaries were younger than FFS beneficiaries (76.7 vs 77.2 years old, *p* < 0.001). Beneficiaries enrolled in MA were more likely to be Black (12.0% vs 7.5%, *p* < 0.001), Asian (1.7% vs 1.5%, *p* < 0.001), and Hispanic (2.5% vs 1.1%, *p *< 0.001), and were less likely to be American Indian or Alaska Native (0.2% vs 0.6%, *p* < 0.001) and White (80.8% vs 86.7%, *p* < 0.001), compared to those enrolled in FFS. MA beneficiaries were also more likely to be dual-eligible for Medicaid coverage (13.7% vs 12.9%, *p* < 0.001). MA beneficiaries were less likely to have three or more Elixhauser comorbidities (82.6% vs 83.1%, *p* = 0.011) and had a lower mean CFI (0.168 vs 0.169, *p* < 0.001), compared to FFS beneficiaries (Table 1).

**Table 1:** Baseline covariate distribution by coverage type

Covariate (*n* (%) unless otherwise noted)	Medicare advantage *n* = 53,023	Fee-for-service *n* = 95,846	*p*-value*
Age, Mean (SD)	76.7 (7.1)	77.2 (7.5)	< 0.001^a^
Sex			0.020
Male	25,444 (48.0)	45,390 (47.4)	
Female	27,579 (52.0)	50,456 (52.6)	
Race			< 0.001
Unknown	566 (1.1)	1246 (1.3)	
White	42,868 (80.8)	83,087 (86.7)	
Black	6351 (12.0)	7154 (7.5)	
Other	935 (1.8)	1322 (1.4)	
Asian	896 (1.7)	1423 (1.5)	
American Indian or Alaska Native	90 (0.2)	545 (0.6)	
Hispanic Ethnicity	1317 (2.5)	1069 (1.1)	< 0.001
Dual-Eligible	7256 (13.7)	12,403 (12.9)	< 0.001
Elixhauser Comorbidity Index			0.011
0	581 (1.1)	918 (1.0)	
1	2777 (5.2)	4823 (5.0)	
2	5890 (11.1)	10,493 (10.9)	
3 +	43,775 (82.6)	79,612 (83.1)	
Individual Elixhauser Comorbidities			
AIDS/HIV	92 (0.2)	131 (0.1)	0.091
Alcohol Abuse	1620 (3.1)	2691 (2.8)	0.007
Deficiency Anemia	11,110 (21.0)	31,538 (32.9)	< 0.001
Rheumatoid Arthritis	3057 (5.8)	5404 (5.6)	0.316
Blood Loss Anemia	2690 (5.1)	7804 (8.1)	< 0.001
Lymphoma	807 (1.5)	1658 (1.7)	0.003
Leukemia	403 (0.8)	809 (0.8)	0.090
Metastatic Cancer	13,285 (25.1)	24,952 (26.0)	< 0.001
Solid Tumor without Metastasis, In Situ	1108 (2.1)	2343 (2.4)	< 0.001
Solid Tumor without Metastasis, Malignant	34,850 (65.7)	61,427 (64.1)	< 0.001
Cerebrovascular Disease	2106 (4.0)	4401 (4.6)	< 0.001
Congestive Heart Failure	4846 (9.1)	13,234 (13.8)	< 0.001
Coagulopathy	1419 (2.7)	4136 (4.3)	< 0.001
Dementia	3608 (6.8)	7476 (7.8)	< 0.001
Depression	8849 (16.7)	14,424 (15.0)	< 0.001
Diabetes Uncomplicated	17,117 (32.3)	28,195 (29.4)	< 0.001
Diabetes Complicated	13,839 (26.1)	19,550 (20.4)	< 0.001
Drug Abuse	1148 (2.2)	983 (1.0)	< 0.001
Hypertension Complicated	17,313 (32.7)	26,437 (27.6)	< 0.001
Hypertension Uncomplicated	41,712 (78.7)	74,498 (77.7)	< 0.001
Liver Disease Moderate	1842 (3.5)	4930 (5.1)	< 0.001
Liver Disease Severe	230 (0.4)	607 (0.6)	< 0.001
Chronic Pulmonary Disease	14,904 (28.1)	25,240 (26.3)	< 0.001
Neurological Disorders affecting Movement	763 (1.4)	2452 (2.6)	< 0.001
Other Neurological Disorders	796 (1.5)	2,439 (2.5)	< 0.001
Seizures and Epilepsy	531 (1.0)	1584 (1.7)	< 0.001
Obesity	15,023 (28.3)	22,773 (23.8)	< 0.001
Paralysis	1175 (2.2)	2398 (2.5)	0.001
Peripheral Vascular Disease	16,106 (30.4)	23,222 (24.2)	< 0.001
Psychoses	512 (1.0)	1326 (1.4)	< 0.001
Pulmonary Circulation Disorder	1360 (2.6)	3743 (3.9)	< 0.001
Renal Failure Moderate	5315 (10.0)	12,281 (12.8)	< 0.001
Renal Failure Severe	1087 (2.1)	2843 (3.0)	< 0.001
Hypothyroidism	11,267 (21.2)	21,268 (22.2)	< 0.001
Other Thyroid Disease	2800 (5.3)	4645 (4.8)	< 0.001
Peptic Ulcer Disease excluding bleeding	768 (1.4)	2068 (2.2)	< 0.001
Valvular Disease	3337 (6.3)	9101 (9.5)	< 0.001
Weight Loss	5285 (10.0)	14,488 (15.1)	< 0.001
Multimorbid	26,427 (49.8)	46,883 (48.9)	< 0.001
Claims-based Frailty Index, Mean (SD)	0.168 (0.06)	0.169 (0.06)	< 0.001
CRC Tumor Location			0.011
C18: Colon	44,283 (83.5)	80,617 (84.1)	
C19: Rectosigmoid	2746 (5.2)	4751 (5.0)	
C20: Rectal	5994 (11.3)	10,478 (10.9)	
CRC Procedure Type			
Partial Colectomy	47,403 (89.4)	85,964 (89.7)	0.082
Total Colectomy	1069 (2.0)	1724 (1.8)	0.003
Large Bowel Procedure	166 (0.3)	271 (0.3)	0.324
Proctectomy	2426 (4.6)	4079 (4.3)	0.004
Multimodal Therapy			
Chemotherapy	3277 (6.2)	6384 (6.7)	< 0.001
Radiation Therapy	3590 (6.8)	6736 (7.0)	0.060
Metastases			
Lymph Node Metastases	6844 (12.9)	13,833 (14.4)	< 0.001
Distant Metastases	8227 (15.5)	16,294 (17.0)	< 0.001
Access Type			0.069
Emergent/Urgent	17,790 (33.6)	31,711 (33.1)	
Elective	35,233 (66.4)	64,135 (66.9)	

*SD* Standard Deviation, *AIDS* Acquired Immunodeficiency Syndrome, *HIV* Human Immunodeficiency Virus, *CRC* Colorectal Cancer

*All *p*-values computed derived via χ^2^ test unless otherwise noted. ^a^*P*-value derived from *T*-test

In terms of CRC characteristics, MA beneficiaries were more likely to undergo resection for rectosigmoid (5.2% vs 5.0%, *p* = 0.011) and rectal cancers (11.3% vs 10.9%, *p* = 0.011), compared to FFS beneficiaries. There was no statistically significant difference in receipt of partial colectomy by coverage type, however MA beneficiaries were more likely to undergo total colectomy (2.0% vs 1.8%, *p* = 0.003) or proctectomy (4.6% vs 4.3%, *p* = 0.004) compared to FFS beneficiaries. Those enrolled in MA were less likely to have lymph node metastases (12.9 vs 14.4%, *p* < 0.001) or distant metastases (15.5% vs 17.0%, *p* < 0.001), compared to FFS beneficiaries. Furthermore, MA beneficiaries were less likely to receive chemotherapy (6.2% vs 6.7%, *p* < 0.001) compared to their FFS counterparts, although there was no difference in receipt of radiation therapy. There was no statistically significant difference with regards to receiving care in the emergent/urgent versus elective setting by coverage type (*p* = 0.069) (Table 1).

In terms of operative approach, MA beneficiaries were more likely to undergo laparoscopic resection compared to FFS beneficiaries (54.7% vs 52.6%, *p* < 0.001). Furthermore, there was no statistically significant difference with regards to robotic assistance by coverage type (MA: 13.8%, FFS: 13.9%, *p* = 0.546).

### Adjusted tapered match outcomes

Table 2 shows the characteristics of MA beneficiaries and FFS beneficiaries before and after weighting sequentially for additional covariates. After tapered specifications employing balancing weights, there were no statistically significant differences in any of the matched covariates between the two groups as seen by standardized mean differences with an absolute value of < 0.001.

**Table 2:** Quality Assessment for Tapered Matches

Covariate (Proportion unless otherwise noted)	Before weighting			Taper 1: Patient Demographics			Taper 2: Socioeconomic Status			Taper 3: Baseline Medical Status			Taper 4: Cancer Characteristics			Taper 5: Access			Taper 6: Hospital		
	MA	FFS	SD	MA	FFS	SD	MA	FFS	SD	MA	FFS	SD	MA	FFS	SD	MA	FFS	SD	MA	FFS	SD
Age (mean years)	76.7	77.2	− 0.1	76.7	76.7	0.0	76.7	76.7	0.0	76.7	76.7	0.0	76.7	76.7	0.0	76.7	76.7	0.0	76.7	76.7	0.0
White Race	0.8	0.9	− 0.2	0.8	0.8	0.0	0.8	0.8	0.0	0.8	0.8	0.0	0.8	0.8	0.0	0.8	0.8	0.0	0.8	0.8	0.0
Black Race	0.1	0.1	0.2	0.1	0.1	0.0	0.1	0.1	0.0	0.1	0.1	0.0	0.1	0.1	0.0	0.1	0.1	0.0	0.1	0.1	0.0
Asian Race	0.0	0.0	0.0	0.0	0.0	0.0	0.0	0.0	0.0	0.0	0.0	0.0	0.0	0.0	0.0	0.0	0.0	0.0	0.0	0.0	0.0
American Indian/Alaska Native	0.0	0.0	0.1	0.0	0.0	0.0	0.0	0.0	0.0	0.0	0.0	0.0	0.0	0.0	0.0	0.0	0.0	0.0	0.0	0.0	0.0
Other Race	0.0	0.0	− 0.1	0.0	0.0	0.0	0.0	0.0	0.0	0.0	0.0	0.0	0.0	0.0	0.0	0.0	0.0	0.0	0.0	0.0	0.0
Unknown Race	0.0	0.0	0.0	0.0	0.0	0.0	0.0	0.0	0.0	0.0	0.0	0.0	0.0	0.0	0.0	0.0	0.0	0.0	0.0	0.0	0.0
Hispanic Ethnicity	0.0	0.0	0.0	0.0	0.0	0.0	0.0	0.0	0.0	0.0	0.0	0.0	0.0	0.0	0.0	0.0	0.0	0.0	0.0	0.0	0.0
Female Sex	0.5	0.5	0.0	0.5	0.5	0.0	0.5	0.5	0.0	0.5	0.5	0.0	0.5	0.5	0.0	0.5	0.5	0.0	0.5	0.5	0.0
Dual Eligibility	0.1	0.1	0.0	0.1	0.1	0.0	0.1	0.1	0.0	0.1	0.1	0.0	0.1	0.1	0.0	0.1	0.1	0.0	0.1	0.1	0.0
0 Elixhauser Comorbidities	0.0	0.0	0.0	0.0	0.0	0.0	0.0	0.0	0.0	0.0	0.0	0.0	0.0	0.0	0.0	0.0	0.0	0.0	0.0	0.0	0.0
1 Elixhauser Comorbidities	0.1	0.1	0.0	0.1	0.1	0.0	0.1	0.1	0.0	0.1	0.1	0.0	0.1	0.1	0.0	0.1	0.1	0.0	0.1	0.1	0.0
2 Elixhauser Comorbidities	0.1	0.1	0.0	0.1	0.1	0.0	0.1	0.1	0.0	0.1	0.1	0.0	0.1	0.1	0.0	0.1	0.1	0.0	0.1	0.1	0.0
3 Elixhauser Comorbidities	0.8	0.8	0.0	0.8	0.8	0.0	0.8	0.8	0.0	0.8	0.8	0.0	0.8	0.8	0.0	0.8	0.8	0.0	0.8	0.8	0.0
CFI	0.2	0.2	0.0	0.2	0.2	0.0	0.2	0.2	0.0	0.2	0.2	0.0	0.2	0.2	0.0	0.2	0.2	0.0	0.2	0.2	0.0
Multimorbidity	0.5	0.5	0.0	0.5	0.5	0.0	0.5	0.5	0.0	0.5	0.5	0.0	0.5	0.5	0.0	0.5	0.5	0.0	0.5	0.5	0.0
CRC Type: Colon	0.8	0.8	0.0	0.8	0.8	0.0	0.8	0.8	0.0	0.8	0.8	0.0	0.8	0.8	0.0	0.8	0.8	0.0	0.8	0.8	0.0
CRC Type: Rectosigmoid	0.1	0.0	0.0	0.1	0.1	0.0	0.1	0.1	0.0	0.1	0.1	0.0	0.1	0.1	0.0	0.1	0.1	0.0	0.1	0.1	0.0
CRC Type: Rectal	0.1	0.1	0.0	0.1	0.1	0.0	0.1	0.1	0.0	0.1	0.1	0.0	0.1	0.1	0.0	0.1	0.1	0.0	0.1	0.1	0.0
Distant Metastases	0.2	0.2	0.0	0.2	0.2	0.0	0.2	0.2	0.0	0.2	0.2	0.0	0.2	0.2	0.0	0.2	0.2	0.0	0.2	0.2	0.0
Lymph Node Metastases	0.1	0.1	0.0	0.1	0.1	0.0	0.1	0.1	0.0	0.1	0.1	0.0	0.1	0.1	0.0	0.1	0.1	0.0	0.1	0.1	0.0
Chemotherapy	0.1	0.1	0.0	0.1	0.1	0.0	0.1	0.1	0.0	0.1	0.1	0.0	0.1	0.1	0.0	0.1	0.1	0.0	0.1	0.1	0.0
Radiation Therapy	0.1	0.1	0.0	0.1	0.1	0.0	0.1	0.1	0.0	0.1	0.1	0.0	0.1	0.1	0.0	0.1	0.1	0.0	0.1	0.1	0.0
Partial Colectomy	0.9	0.9	0.0	0.9	0.9	0.0	0.9	0.9	0.0	0.9	0.9	0.0	0.9	0.9	0.0	0.9	0.9	0.0	0.9	0.9	0.0
Total Colectomy	0.0	0.0	0.0	0.0	0.0	0.0	0.0	0.0	0.0	0.0	0.0	0.0	0.0	0.0	0.0	0.0	0.0	0.0	0.0	0.0	0.0
Large Bowel Procedure	0.0	0.0	0.0	0.0	0.0	0.0	0.0	0.0	0.0	0.0	0.0	0.0	0.0	0.0	0.0	0.0	0.0	0.0	0.0	0.0	0.0
Proctectomy	0.0	0.0	0.0	0.0	0.0	0.0	0.0	0.0	0.0	0.0	0.0	0.0	0.0	0.0	0.0	0.0	0.0	0.0	0.0	0.0	0.0
Ostomy	0.1	0.1	0.0	0.1	0.1	0.0	0.1	0.1	0.0	0.1	0.1	0.0	0.1	0.1	0.0	0.1	0.1	0.0	0.1	0.1	0.0
Emergent/Urgent Admission	0.3	0.3	0.0	0.3	0.3	0.0	0.3	0.3	0.0	0.3	0.3	0.0	0.3	0.3	0.0	0.3	0.3	0.0	0.3	0.3	0.0

*MA* Medicare Advantage, *FFS* Fee-for-Service, *SD* Standardized Difference, *CFI* Claims-Based Frailty Index, *CRC* Colorectal Cancer

MA beneficiaries had a higher likelihood of undergoing laparoscopic CRC resection before weighting (OR 1.12 (1.09–1.14), *p* < 0.001), and after weighting for all additional covariates (OR 1.12 (1.10–1.15), *p* < 0.001), compared to FFS beneficiaries. On the other hand, there was no statistically significant difference in the use of robotic assistance by enrollment plan, both before weighting (OR 1.00 (0.97–1.04), *p* = 0.800) and after weighting (OR 1.00 (0.97–1.03), *p* = 0.912) (Table 3).

**Table 3:** Operative Approach Outcomes for Medicare Advantage Beneficiaries (Reference Group: Medicare Fee-for-Service Beneficiaries)

Outcome	Cohort	Odds ratio (95% Confidence interval)	*p*-value
Laparoscopic Approach	Before weighting	1.12 (1.09–1.14)	< 0.001
	Patient demographics	1.12 (1.09–1.14)	< 0.001
	Patient Demographics + Socioeconomic Status	1.12 (1.09–1.14)	< 0.001
	Patient Demographics + Socioeconomic Status + Baseline Medical Status	1.12 (1.10–1.15)	< 0.001
	Patient Demographics + Socioeconomic Status + Baseline Medical Status + Cancer Characteristics	1.11 (1.09–1.14)	< 0.001
	Patient Demographics + Socioeconomic Status + Baseline Medical Status + Cancer Characteristics + Access	1.12 (1.10–1.15)	< 0.001
	Patient Demographics + Socioeconomic Status + Baseline Medical Status + Cancer Characteristics + Access + Hospital	1.12 (1.10–1.15)	< 0.001
Robotic Assistance	Before weighting	1.00 (0.97–1.04)	0.800
	Patient demographics	0.99 (0.96–1.03)	0.752
	Patient Demographics + Socioeconomic Status	0.99 (0.96–1.02)	0.671
	Patient Demographics + Socioeconomic Status + Baseline Medical Status	1.00 (0.97–1.03)	0.822
	Patient Demographics + Socioeconomic Status + Baseline Medical Status + Cancer Characteristics	0.99 (0.96–1.03)	0.697
	Patient Demographics + Socioeconomic Status + Baseline Medical Status + Cancer Characteristics + Access	1.00 (0.97–1.03)	0.982
	Patient Demographics + Socioeconomic Status + Baseline Medical Status + Cancer Characteristics + Access + Hospital	1.00 (0.97–1.03)	0.912

There was no significant difference in hospital length-of-stay by enrollment plan, both before weighting (*β* coefficient 0.02 (− 0.06–0.10), *p* = 0.602) and after weighting for all additional covariates (*β* coefficient 0.03 (− 0.05–0.10), *p* = 0.461) (Table 4).

**Table 4:** Hospital Length-of-Stay Outcomes for Medicare Advantage Beneficiaries (Reference Group: Medicare Fee-for-Service Beneficiaries)

Outcome	Cohort	Point estimate (95% Confidence interval)	*p*-value
Hospital Length-of-Stay	Before Weighting	0.02 (− 0.06–0.10)	0.602
	Patient Demographics	0.03 (− 0.04–0.11)	0.415
	Patient Demographics + Socioeconomic Status	0.05 (− 0.03–0.12)	0.208
	Patient Demographics + Socioeconomic Status + Baseline Medical Status	0.03 (− 0.05–0.10)	0.464
	Patient Demographics + Socioeconomic Status + Baseline Medical Status + Cancer Characteristics	0.04 (− 0.03–0.12)	0.289
	Patient Demographics + Socioeconomic Status + Baseline Medical Status + Cancer Characteristics + Access	0.01 (− 0.07–0.08)	0.812
	Patient Demographics + Socioeconomic Status + Baseline Medical Status + Cancer Characteristics + Access + Hospital	0.03 (− 0.05–0.10)	0.461

Before weighting, MA beneficiaries had lower odds of 30-day mortality (OR 0.94 (0.90–0.98), *p* = 0.008), 60-day mortality (OR 0.94 (0.90–0.97), *p* = 0.001), and 90-day mortality (OR 0.92 (0.89–0.96), *p* < 0.001), compared to FFS beneficiaries. However, differences in mortality rates within 30-days, 60-days, and 90-days were no longer significant after weighting for patient demographics (OR 0.99 (0.94–1.03), *p* = 0.571; OR 0.98 (0.94–1.02), *p* = 0.302; and OR 0.96 (0.93–1.00), *p* = 0.055, respectively) (Table 5).

**Table 5:** Mortality Outcomes for Medicare Advantage Beneficiaries (Reference Group: Medicare Fee-for-Service Beneficiaries)

Outcome	Cohort	Odds ratio (95% Confidence interval)	*p*-value
30-Day Mortality	Before Weighting	0.94 (0.90–0.98)	0.008
	Patient Demographics	0.99 (0.94–1.03)	0.571
	Patient Demographics + Socioeconomic Status	0.99 (0.95–1.04)	0.736
	Patient Demographics + Socioeconomic Status + Baseline Medical Status	0.99 (0.94–1.03)	0.549
	Patient Demographics + Socioeconomic Status + Baseline Medical Status + Cancer Characteristics	1.00 (0.96–1.05)	0.898
	Patient Demographics + Socioeconomic Status + Baseline Medical Status + Cancer Characteristics + Access	1.00 (0.95–1.04)	0.859
	Patient Demographics + Socioeconomic Status + Baseline Medical Status + Cancer Characteristics + Access + Hospital	0.99 (0.95–1.04)	0.787
60-Day Mortality	Before Weighting	0.94 (0.90–0.97)	0.001
	Patient Demographics	0.98 (0.94–1.02)	0.302
	Patient Demographics + Socioeconomic Status	0.98 (0.94–1.03)	0.442
	Patient Demographics + Socioeconomic Status + Baseline Medical Status	0.98 (0.94–1.02)	0.289
	Patient Demographics + Socioeconomic Status + Baseline Medical Status + Cancer Characteristics	1.00 (0.96–1.04)	0.897
	Patient Demographics + Socioeconomic Status + Baseline Medical Status + Cancer Characteristics + Access	1.00 (0.96–1.04)	0.853
	Patient Demographics + Socioeconomic Status + Baseline Medical Status + Cancer Characteristics + Access + Hospital	1.00 (0.96–1.04)	0.815
90-Day Mortality	Before Weighting	0.92 (0.89–0.96)	< 0.001
	Patient Demographics	0.96 (0.93–1.00)	0.055
	Patient Demographics + Socioeconomic Status	0.97 (0.93–1.01)	0.101
	Patient Demographics + Socioeconomic Status + Baseline Medical Status	0.96 (0.93–1.00)	0.055
	Patient Demographics + Socioeconomic Status + Baseline Medical Status + Cancer Characteristics	0.99 (0.95–1.03)	0.616
	Patient Demographics + Socioeconomic Status + Baseline Medical Status + Cancer Characteristics + Access	0.98 (0.95–1.02)	0.414
	Patient Demographics + Socioeconomic Status + Baseline Medical Status + Cancer Characteristics + Access + Hospital	0.98 (0.95–1.02)	0.380

Furthermore, MA beneficiaries had a lower likelihood of non-routine disposition, both before weighting (OR 0.74 (0.73–0.76), *p* < 0.001) and after weighting for all additional covariates (OR 0.77 (0.75–0.78), *p* < 0.001), compared to FFS beneficiaries (Table 6).

**Table 6:** Disposition Outcomes for Medicare Advantage Beneficiaries (Reference Group: Medicare Fee-for-Service Beneficiaries)

Outcome	Cohort	Odds ratio (95% Confidence interval)	*p*-value
Non-Routine Disposition	Before Weighting	0.74 (0.73–0.76)	< 0.001
	Patient Demographics	0.76 (0.74–0.78)	< 0.001
	Patient Demographics + Socioeconomic Status	0.77 (0.75–0.78)	< 0.001
	Patient Demographics + Socioeconomic Status + Baseline Medical Status	0.77 (0.75–0.78)	< 0.001
	Patient Demographics + Socioeconomic Status + Baseline Medical Status + Cancer Characteristics	0.77 (0.75–0.79)	< 0.001
	Patient Demographics + Socioeconomic Status + Baseline Medical Status + Cancer Characteristics + Access	0.76 (0.75–0.78)	< 0.001
	Patient Demographics + Socioeconomic Status + Baseline Medical Status + Cancer Characteristics + Access + Hospital	0.77 (0.75–0.78)	< 0.001

Lastly, MA beneficiaries had an overall lower likelihood of readmission, compared to FFS beneficiaries within 30-days (before weighting OR 0.78 [0.75–0.81], *p* < 0.001, after weighting OR 0.76 [0.73–0.80], *p* < 0.001), 60-days (before weighting OR 0.80 [0.77–0.83], *p* < 0.001, after weighting OR 0.79 [0.76–0.82], *p* < 0.001), and 90-days (before weighting OR 0.79 [0.77–0.82], *p* < 0.001, after weighting OR 0.79 [0.76–0.81], *p* < 0.001) (Table 7).

**Table 7:** Readmission Outcomes for Medicare Advantage Beneficiaries (Reference Group: Medicare Fee-for-Service Beneficiaries)

Outcome	Cohort	Odds ratio (95% Confidence interval)	*p*-value
30-Day Readmission	Before Weighting	0.78 (0.75–0.81)	< 0.001
	Patient Demographics	0.77 (0.74–0.80)	< 0.001
	Patient Demographics + Socioeconomic Status	0.77 (0.74–0.80)	< 0.001
	Patient Demographics + Socioeconomic Status + Baseline Medical Status	0.77 (0.74–0.80)	< 0.001
	Patient Demographics + Socioeconomic Status + Baseline Medical Status + Cancer Characteristics	0.76 (0.73–0.79)	< 0.001
	Patient Demographics + Socioeconomic Status + Baseline Medical Status + Cancer Characteristics + Access	0.76 (0.73–0.79)	< 0.001
	Patient Demographics + Socioeconomic Status + Baseline Medical Status + Cancer Characteristics + Access + Hospital	0.76 (0.73–0.80)	< 0.001
60-Day Readmission	Before Match	0.80 (0.77–0.83)	< 0.001
	Patient Demographics	0.79 (0.76–0.82)	< 0.001
	Patient Demographics + Socioeconomic Status	0.79 (0.76–0.82)	< 0.001
	Patient Demographics + Socioeconomic Status + Baseline Medical Status	0.79 (0.76–0.82)	< 0.001
	Patient Demographics + Socioeconomic Status + Baseline Medical Status + Cancer Characteristics	0.79 (0.76–0.82)	< 0.001
	Patient Demographics + Socioeconomic Status + Baseline Medical Status + Cancer Characteristics + Access	0.78 (0.75–0.81)	< 0.001
	Patient Demographics + Socioeconomic Status + Baseline Medical Status + Cancer Characteristics + Access + Hospital	0.79 (0.76–0.82)	< 0.001
90-Day Readmission	Before Weighting	0.79 (0.77–0.82)	< 0.001
	Patient Demographics	0.79 (0.76–0.81)	< 0.001
	Patient Demographics + Socioeconomic Status	0.79 (0.76–0.82)	< 0.001
	Patient Demographics + Socioeconomic Status + Baseline Medical Status	0.79 (0.76–0.82)	< 0.001
	Patient Demographics + Socioeconomic Status + Baseline Medical Status + Cancer Characteristics	0.78 (0.76–0.81)	< 0.001
	Patient Demographics + Socioeconomic Status + Baseline Medical Status + Cancer Characteristics + Access	0.78 (0.75–0.81)	< 0.001
	Patient Demographics + Socioeconomic Status + Baseline Medical Status + Cancer Characteristics + Access + Hospital	0.79 (0.76–0.81)	< 0.001

## Discussion

This study examines the differences in utilization of minimally invasive surgery for colorectal cancer (CRC), as well as postoperative outcomes, among Medicare Advantage (MA) and Medicare Fee-for-Service (FFS) beneficiaries. Compared to FFS beneficiaries, MA beneficiaries were more likely to undergo laparoscopic CRC resection, more likely to be discharged to home, and less likely to be readmitted postoperatively. Notably, there was no difference in the rates of robotic assistance or mortality by Medicare enrollment plan.

MA beneficiaries were more likely to undergo CRC resection via laparoscopic approach compared to FFS beneficiaries. It is possible that MA beneficiaries were less likely to be converted to open operations in the setting of less metastatic spread, which has been found to be an independent factor of conversion to open for CRC operations [37]. The use of minimally invasive techniques for CRC is met with a high learning curve [38], thus approach is often selected based on surgeon expertise and comfort level and MA beneficiaries may be treated by surgeons that more frequently utilize laparoscopic techniques. It is also possible that unaccounted patient-related factors such as prior abdominal surgeries influenced the decision on operative approach.

Furthermore, the study found no difference in the use of robotic assistance by enrollment plan. This suggests that MA beneficiaries have access to providers that use new and expensive technology, despite their narrow network coverage [39]. Additionally, similar rates of mortality among MA and FFS beneficiaries suggest similar short-term quality of CRC surgical care among those who qualify for surgical intervention. This is supported by a recent study by Raoof et al. that found MA beneficiaries have similar access to high-volume hospitals for CRC surgical care, compared to traditional Medicare beneficiaries [40].

MA beneficiaries were more likely to be discharged to home postoperatively, and less likely to be readmitted. One explanation for this finding is that capitated payments for total cost of care made to MA plans incentivize methods to limit costs, including avoiding unnecessary post-acute care [13]. Our findings are consistent with previous studies that found MA beneficiaries were less likely to use post-acute care or have shorter stays at nursing facilities, but also had lower likelihood of hospital readmission and higher rates of return to community, compared to FFS beneficiaries [19, 41].

The results of this study indicate that compared to FFS beneficiaries, MA beneficiaries were less likely to have metastases or to receive chemotherapy. Although we are unable to discern cancer stage in this study, these findings suggest that MA beneficiaries may experience earlier CRC detection compared to FFS beneficiaries. This is supported by previous literature that consistently shows that MA enrollees have higher rates of utilization of preventative services compared to traditional Medicare enrollees [42–44]. Furthermore, several studies report higher rates of CRC screenings in beneficiaries enrolled in MA compared to FFS [44, 45]. This may be explained by MA plan reimbursement models that incentivize high quality care or could be due to better care coordination in the setting of narrower provider networks that share electronic health networks [44]. Additionally, navigation support for MA beneficiaries in the form of appointment reminders and transportation assistance may improve the ability to schedule and complete cancer screenings [46].

There are several limitations to this study. First, this is a retrospective cohort study of Medicare beneficiaries which limits the generalizability of our findings. Second, we are unable to discern operations that started with an open approach from those that were converted to open from laparoscopic or robotic techniques [47]. However, if conversion rates were greater in the FFS group, then our effect size would be even greater. If conversion rates were greater in the MA group, it would bias us towards the null. Third, although the database does provide information regarding metastases and use of medical therapies, it does not provide complete cancer stage information and therefore the number of locally invasive tumors is unknown [48, 49]. Furthermore, we are unable to determine the number of patients who underwent total neoadjuvant therapy for rectal cancer specifically, limiting our ability to draw conclusions regarding surgical treatment for recurrence. Fourth, as we are unable to discern location of rectal tumors, we are limited in our ability to adjust for type of proctectomy performed and how it impacts operative technique and outcomes. However, these are real patient data that reflect the standard of care in the modern era as determined by practicing surgeons. Further, the proportion of abdominoperineal resection to low anterior resection is 1:3 to 1:4 and unlikely to differ between MA and FFS patients, thus unlikely to impact our findings [50, 51]. Lastly, this study only includes beneficiaries who underwent operative treatment. As such, it is possible that surgeon selection of operative candidates may differ by enrollment plan [52].

In summary, MA beneficiaries are more likely to undergo laparoscopic CRC resection compared to FFS beneficiaries, which may be due to ease of operability in the setting of less cancer spread. Furthermore, compared to FFS beneficiaries, MA beneficiaries receive similar quality CRC surgical care with regards to short-term morbidity and mortality, and recover at home.

## Supplementary Information

Below is the link to the electronic supplementary material.

Supplementary file1 (DOCX 21 KB)
