# Supplementary material for: Minimally invasive colorectal cancer surgery: an observational study of medicare advantage and fee-for-service beneficiaries
Source: Surg Endosc. 2024 Aug 19;38(11):6800–11. doi: 10.1007/s00464-024-11168-0 (PMC11525327; doi:10.1007/s00464-024-11168-0)
Supplement: Supplementary file 1 — Supplementary file1 (DOCX 21 KB) [file 464_2024_11168_MOESM1_ESM.docx]

Supplement Table 1. Colorectal Cancer ICD-10 Diagnosis Codes

| **Code** | **Description** | **Cancer Type** |
| --- | --- | --- |
| C180 | Malignant neoplasm of cecum | Colon |
| C181 | Malignant neoplasm of appendix | Colon |
| C182 | Malignant neoplasm of ascending colon | Colon |
| C183 | Malignant neoplasm of hepatic flexure | Colon |
| C184 | Malignant neoplasm of transverse colon | Colon |
| C185 | Malignant neoplasm of splenic flexure | Colon |
| C186 | Malignant neoplasm of descending colon | Colon |
| C187 | Malignant neoplasm of sigmoid colon | Colon |
| C188 | Malignant neoplasm of overlapping sites of colon | Colon |
| C189 | Malignant neoplasm of colon, unspecified | Colon |
| C19 | Malignant neoplasm of rectosigmoid junction | Rectosigmoid |
| C20 | Malignant neoplasm of rectum | Rectal |

Supplement Table 2. Colorectal Cancer Procedure CPT Codes

| **CPT Code** | **CPT Label** | **Procedure Type** | **Operative Approach** |
| --- | --- | --- | --- |
| 44204 | Laparoscopy, surgical; colectomy, partial, with anastomosis | Partial Colectomy | Laparoscopic |
| 44205 | Laparoscopy, surgical; colectomy, partial, with removal of terminal ileum with ileocolostomy | Partial Colectomy | Laparoscopic |
| 44206 | Laparoscopy, surgical; colectomy, partial with end colostomy and closure of distal segment (Hartmann) | Partial Colectomy | Laparoscopic |
| 44207 | Laparoscopy, surgical; colectomy, partial, with anastomosis, with coloproctostomy (low pelvic anastomosis) | Partial Colectomy | Laparoscopic |
| 44208 | Laparoscopy, surgical; colectomy, partial, with anastomosis, with coloproctostomy (low pelvic anastomosis) with colostomy | Partial Colectomy | Laparoscopic |
| 44130 | Enteroenterostomy, anastomosis of intestine, with or without cutaneous enterostomy (separate procedure) | Partial Colectomy | Open |
| 44140 | Colectomy, partial; with anastomosis | Partial Colectomy | Open |
| 44141 | Colectomy, partial; with skin level cecostomy or colostomy | Partial Colectomy | Open |
| 44143 | Colectomy, partial; with end colostomy and closure of distal segment | Partial Colectomy | Open |
| 44144 | Colectomy, partial; with colostomy or ileostomy and creation of mucofistula | Partial Colectomy | Open |
| 44145 | Colectomy, partial; with coloproctostomy (low pelvic anastomosis) | Partial Colectomy | Open |
| 44146 | Colectomy, partial; with coloproctostomy (low pelvic anastomosis) with colostomy | Partial Colectomy | Open |
| 44147 | Colectomy, partial; abdominal and transanal approach | Partial Colectomy | Open |
| 44160 | Colectomy, partial, with removal of terminal ileum with ileocolostomy | Partial Colectomy | Open |
| 44625 | Closure of enterostomy, large or small intestine; with resection and anastomosis other than colorectal | Partial Colectomy | Open |
| 44626 | Closure of enterostomy, large or small intestine; with resection and colorectal anastomosis (eg, closure of Hartmann type procedure) | Partial Colectomy | Open |
| 44210 | Laparoscopy, surgical; colectomy, total, abdominal, without proctectomy, with ileostomy or ileoproctostomy | Total Colectomy | Laparoscopic |
| 44211 | Laparoscopy, surgical; colectomy, total, abdominal, with proctectomy, with ileoanal anastomosis, creation of ileal reservoir | Total Colectomy | Laparoscopic |
| 44212 | Laparoscopy, surgical; colectomy, total, abdominal, with proctectomy, with ileostomy | Total Colectomy | Laparoscopic |
| 44150 | Colectomy, total, abdominal, without proctectomy; with ileostomy or ileoproctostomy | Total Colectomy | Open |
| 44151 | Colectomy, total, abdominal without proctectomy; with continent ileostomy | Total Colectomy | Open |
| 44152 | Colectomy, total, abdominal, without proctectomy; with rectal mucosectomy, ileoanal anastomosis, with or without loop ileostomy | Total Colectomy | Open |
| 44153 | Colectomy, total, abdominal, without proctectomy; with rectal mucosectomy, ileoanal anastomosis, creation of ileal reservoir (S or J), with or without loop ileostomy | Total Colectomy | Open |
| 44155 | Colectomy, total, abdominal, with proctectomy; with ileostomy | Total Colectomy | Open |
| 44156 | Colectomy, total, abdominal, with proctectomy; with continent ileostomy | Total Colectomy | Open |
| 44157 | Colectomy, total, abdominal, with proctectomy; with ileoanal anastomosis | Total Colectomy | Open |
| 44158 | Colectomy, total, abdominal, with proctectomy; with ileoanal anastomosis, creation of ileal reservoir | Total Colectomy | Open |
| 44025 | Colotomy, for exploration, biopsy(s), or foreign body removal | Large Bowel | Open |
| 44130 | Enteroenterostomy, anastomosis of intestine, with or without cutaneous enterostomy (separate procedure) | Large Bowel | Open |
| 44140 | Colectomy, partial; with anastomosis | Large Bowel | Open |
| 44145 | Colectomy, partial; with coloproctostomy (low pelvic anastomosis) | Large Bowel | Open |
| 44160 | Colectomy, partial, with removal of terminal ileum with ileocolostomy | Large Bowel | Open |
| 44205 | Laparoscopy, surgical; colectomy, partial, with removal of terminal ileum with ileocolostomy | Large Bowel | Laparoscopic |
| 44320 | Colostomy or skin level cecostomy; | Large Bowel | Open |
| 44604 | Suture of large intestine (colorrhaphy) for perforated ulcer, diverticulum, wound, injury or rupture (single or multiple perforations); without colostomy | Large Bowel | Open |
| 44605 | Suture of large intestine for perforated ulcer, diverticulu, wound, injury, or rupture; with colostomy | Large Bowel | Open |
| 45130 | Excision of rectal procidentia, with anastomosis; perineal approach | Large Bowel | Open |
| 45135 | Excision of rectal procidentia, with anastomosis; abdominal and perineal approach | Large Bowel | Open |
| 44143 | Colectomy, partial; with end colostomy and closure of distal segment (Hartmann type procedure) | Proctectomy | Open |
| 44145 | Colectomy, partial; with coloproctostomy (low pelvic anastomosis) | Proctectomy | Open |
| 44146 | Colectomy, partial; with coloproctostomy (low pelvic anastomosis), with colostomy | Proctectomy | Open |
| 44147 | Colectomy, partial; abdominal and transanal approach | Proctectomy | Open |
| 44155 | Colectomy, total, abdominal, with proctectomy; with ileostomy | Proctectomy | Open |
| 44207 | Laparoscopy, surgical; colectomy, partial, with anastomosis, with coloproctostomy (low pelvic anastomosis) | Proctectomy | Laparoscopic |
| 45110 | Proctectomy; complete, combined abdominoperineal, with colostomy | Proctectomy | Open |
| 45111 | Proctectomy; partial resection of rectum, transabdominal approach | Proctectomy | Open |
| 45112 | Proctectomy, combined abdominoperineal, pull-through procedure (eg, colo-anal anastomosis) | Proctectomy | Open |
| 45113 | Proctectomy, partial, with rectal mucosectomy, ileoanal anastomosis, creation of ileal reservoir (S or J), with or without loop ileostomy | Proctectomy | Open |
| 45119 | Proctectomy, combined abdominoperineal pull-through procedure (eg, colo-anal anastomosis), with creation of colonic reservoir (eg, J-pouch), with diverting enterostomy when performed | Proctectomy | Open |
| 45120 | Proctectomy, complete, abdominal and perineal approach; with pull-through procedure and anastomosis | Proctectomy | Open |
| 45121 | Proctectomy, complete, abdominal and perineal approach; with subtotal or total colectomy | Proctectomy | Open |
| 45123 | Proctectomy, partial, without anastomosis, perineal approach | Proctectomy | Open |
| 45130 | Excision of rectal procidentia, with anastomosis; perineal approach | Proctectomy | Open |
| 45395 | Laparoscopy, surgical; proctectomy, complete, combined abdominoperineal, with colostomy | Proctectomy | Laparoscopic |
| 45397 | Laparoscopy, surgical; proctectomy, combined abdominoperineal pull-through procedure, with colonic reservoir | Proctectomy | Laparoscopic |
